# Determinants of Antenatal Healthcare Services Utilisation: A Case of Dodoma, Tanzania

**DOI:** 10.24248/eahrj.v6i2.701

**Published:** 2022-11-30

**Authors:** Saraphina J Kibesa, Yona W Kitua, Daniel W Kitua

**Affiliations:** a Institute of Rural Development Planning, Dodoma, Tanzania; b University of Iringa, Iringa, Tanzania; c Muhimbili University of Health and Allied Sciences, Dar-es-Salaam, Tanzania

## Abstract

**Background:**

Antenatal Care (ANC) coverage is a key determinant of maternal and perinatal morbidity and mortality. Low utilization of ANC services and high Maternal Mortality Ratio (MMR) have been reported in the East African Region. Due to the paucity of information on the determinants of ANC utilization in this region, we conducted the study aiming at exploring factors influencing the utilization of ANC services. We further sought opinions that will aid the improvement of utilization of ANC services.

**Methods:**

A triangulation mixed-method study was conducted in August 2021 among forty-five women and ten healthcare providers in a selected health center located in Dodoma Urban District, Tanzania. Information was gathered using semi-structured questionnaires and in-depth interviews. Quantitative data were analysed using IBM SPSS Statistics. The relationship between the outcome variable and the predictor variables was assessed by either the Chi-square test or Fisher's exact test and a *p value<.05* was considered statistically significant. Manual thematic analysis was used for qualitative data after thorough transcript and documentary reviews.

**Results:**

Almost half (48.9%) of the interviewed women attended ANC services at least once during their last pregnancy. Women who reported having a low income and those who spent more than an hour reaching the health facility had poor ANC attendance (*p value<.05*). The main themed factors that negatively impacted ANC utilization included cultural practices and gender norms, poor communication between partners, and long waiting time at the ANC clinics.

**Conclusion:**

Utilization of ANC services was found to be low among women living in Dodoma Urban District. ANC attendance varied with the level of income and the time women spent reachingt the health facility. Cultural practices and gender norms, communication between spouses, and service waiting time were mentioned to influence ANC attendance.

**Recommendations:**

Public and private sectors should invest in maternal health, provide affordable services and formulate strategies to improve the accessibility of ANC services. Interventions should target women of low socio-economic class and those living in remote areas. Moreover, schemes to address the sociocultural barriers to ANC utilization need to be formulated.

## BACKGROUND

**FIGURE 1:**
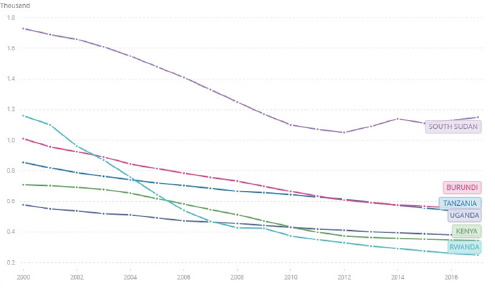
Figure 1. Maternal mortality ratio (modeled estimate, per 100,000 live births) for Tanzania, Kenya, Uganda, Burundi, Rwanda, and South Sudan Adapted from WHO et al.^2^

Despite the average annual decline (2.9%) in the Maternal Mortality Ratio (MMR) in the Sub-Saharan Africa region through the years 2000 to 2015, the region reported the highest estimates (987 maternal deaths per 100,000 live births) among all the ‘Sustainable Development Goals (SDGs)' Regions.^1^ The trend has been more or less similar for the East African Community (EAC) member States (Figure 1) with the highest MMR (1,150 maternal deaths per 100,000 live births) reported in South Sudan as of the year 2017.^2^ A 10-year retrospective study by Bwana et al^3^ conducted across 34 public hospitals in Tanzania reported a rising trend of the in-hospital MMR; from 40.2 to 57.9 per 100,000 live births from the years 2006 to 2015 respectively. Majority of deaths (83.8%) were attributed to direct causes such as eclampsia, obstetric hemorrhage, and maternal sepsis; whereas, anemia and cardiovascular disorders were the main indirect causes.^3^

Antenatal Care (ANC) coverage is vital in reducing potential risks for maternal and perinatal mortality and remains integral for the well-being of the mother and the newborn.^4^ Receiving at least 8 antenatal visits as recommended by the World Health Organization (WHO) increases the chances of detecting and managing potential pregnancy complications as well as enhancing birth preparedness and complication readiness.^5^ Although numerous initiatives have been implemented to improve maternal healthcare coverage, there is still less than average coverage and low utilization of these services, especially in developing countries where approximately 99% of maternal deaths occur.^6,7^

About 59% of pregnant women in Sub-Saharan African countries who attended ANC services met the previous minimum recommendation of four visits; with the highest attendance reported in the Southern Region (78.9%) and the lowest in the Eastern Region (53.4%).^8^ In Tanzania, the utilization of ANC services has been similar to the East Africa regional estimates (56.4%) with relatively lesser utilization reported in rural areas (39.1%) as compared to the urban areas (54.8%).^9,10^ Diverse factors influencing the utilization of ANC services in sub-Saharan Africa have been identified. These include religion, residency, literacy level, economic status, occupation, women's healthcare decision autonomy, media exposure, access to healthcare, and birth order.^9,11–13^

An increase in demand for Maternal and Child Health (MCH) services has been observed following the shift of capital functions from Dar es Salaam to Dodoma at the end of the year 2020. Moreover, Dodoma region has one of the country's highest MMR (417 deaths per 100,000 live births)^14^, and information on the determinants of ANC services utilization in this region is limited. Identifying the determinants of ANC utilization will be essential in informing strategic interventions which will contribute to the reduction of MMR in the region. This study therefore aimed at determining factors that influence the utilization of ANC services in Dodoma, Tanzania, and provides recommendations for improving the utilization of ANC services.

## METHODOLOGY

### Study Area, Period and Design

**FIGURE 2:**
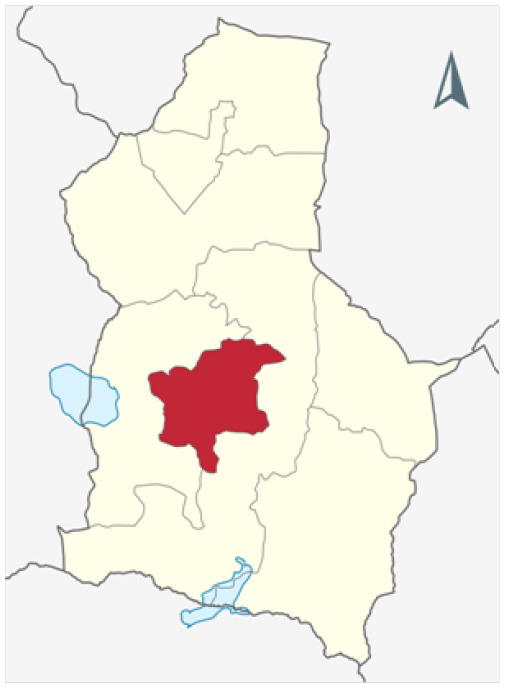
The Map Indicating the Study Area (Dodoma Urban District) in Dodoma Region, Tanzania Adopted from: Nord NordWest (2022)^17^

This study was conducted in a randomly selected health center located in Dodoma Urban District (Figure 2), one of the 8 districts of Dodoma Region in Tanzania. A cross-sectional study design that employed a triangulation mixed-method approach of data collection was used. The method allowed us to compare and interlink findings from quantitative and qualitative data. Furthermore, the design allowed us to examine the major attributes derived from the sets of data and provided a detailed elaboration on the patterns of relationship within the sets of data.

In Tanzania, majority of the ANC services including hospital deliveries take place in dispensaries and health centers.^15^ Health centers are equipped to provide services within a catchment of up to 50,000 people and attend referral cases from dispensaries within the respective catchment area.^16^

### Selection of Participants

A purposive sampling technique was employed in selecting 55 respondents that included 10 healthcare providers (3 doctors, 4 nurses, and 3 medical attendants), and 45 women who resided in Dodoma Region during their antenatal period and gave birth in facilities located in Dodoma. The ‘45 women' were selected from those attending postnatal care at the selected center during the data collection period. A document review was performed to confirm the aforementioned inclusion criteria. The involvement of healthcare providers and postpartum women allowed an in-depth and multi-faceted understanding of the factors which influence the utilization of ANC in the selected health facility.

### Data Collection and Management

Semi-structured questionnaires were used to collect quantitative data, whereas in-depth interview guides were used to collect qualitative data. Both quantitative and qualitative information on the determinants of ANC utilization were collected from the enrolled postpartum women. Saturation of information from the in-depth interview was reached after interviewing 31 women. Opinions on improving ANC utilization were sought from both the healthcare providers and the postpartum women so as to generate care provider- and patient-centered methodological solutions.

Verbatim transcription and translation into English language from the local language were performed for the collected qualitative information. Each questionnaire and transcript was reviewed for content and completeness. All data was securely stored electronically.

### Data Analysis

Quantitative data were analyzed using IBM SPSS Statistics, Version 26.0. Armonk, NY. Descriptive data were presented as frequencies and proportions. Chi-square test or Fisher's exact where applicable was used to assess the relationship between the outcome variable and the predictor variables. A *p value<.05* was considered significant.

Manual thematic analysis approach as proposed by Nowell et al.^18^ was used in qualitative analysis. This included a thorough review of the data followed by thematic coding using the emergent coding approach. Themes were then reviewed and defined prior to the production of the final results.

### Ethical Considerations

Permission to conduct the study was obtained from The Institute of Rural Development Planning (Ref: T40/6 CHRO 33), and the permit to collect data was obtained from Dodoma City Council (Ref: HMD/I.10/6/40). Written informed consent was sought from all the study participants. Information was collected in a secured room and the database was anonymized by coding the participants' identities to ensure confidentiality.

## RESULTS

### Socio-demographic characteristics

A total of 45 women were enrolled in our study. Table 1 shows that majority (60%) of the participants were aged between 18 and 30 years. About two-thirds (60%) of the participants were not married, whereas 8.9% were divorced/separated. Among the respondents, 48.9% and 42.2% were Muslims and Christians respectively. Majority of the participants self-reported a low (51.1%) and middle (46.7%) level of income. The assessment of income was done subjectively (self-reported levels) thus limiting the determination of the cut-point values. Nonetheless, there has been a significant incongruence in the subjective and objective adequacy levels in terms of income making this assessment acceptable.^19^ Among the interviewed women, ANC attendance during the last pregnancy was reported by 48.9%.

**TABLE 1:** Demographics and determinants of ANC Clinic Attendance

Variables	Frequency (%)	Attended ANC	No	P value
Yes	No	
**Age**				
18-30	27 (60.0)	13	14	0.364
31-40	16 (35.6)	8	8	
41-50	2 (4.4)	2	0	
**Marital status**				
Married	14 (31.1)	9	5	0.293
Not married	27 (60.0)	12	15	
Separated/Divorced	4 (8.9)	1	3	
**Education level**				
Primary	11 (24.4)	4	7	0.271
Secondary	22 (48.9)	11	11	
Technical/vocational training	9 (20.0)	4	5	
University/college	3 (6.7)	3	0	
**Religion**				
Christian	19 (42.2)	13	6	0.074
Muslim	22 (48.9)	8	14	
No Religion	4 (8.9)	1	3	
**Self-reported level of income**				
Low income	23 (51.1)	6	17	0.006
Middle income	21 (46.7)	15	6	
High income	1 (2.2)	1	0	
**Time to reach the health facility**				
< 30 minutes	9 (20.0)	8	1	0.026
30 minutes - 1 hour	22 (48.9)	9	13	
> 1 hour	14 (31.1)	5	9	
**Means of transport**				
Public buses	22 (48.9)	8	14	0.136
Motorbike	11 (24.4)	7	4	
Three-wheelers	9 (20.0)	4	5	
On foot	3 (6.7)	3	0	
**Awareness of ANC services**				
Aware	41 (91.1)	21	20	0.608
Not aware	4 (8.9)	1	3	

ANC = Antenatal Care

### Quantitative results of factors influencing utilization of ANC services

As depicted in Table 1, about half (48.9%) of respondents spent between 30 minutes and 1 hour to reach the health facility. However, a third of the respondents (31%) spent more than an hour reaching the health facility. The commonest means of transport to the health facilities were public buses. Despite high awareness of ANC services (91%), utilization of the services was low.

Self-reported level of income and time taken to reach the health facility significantly influenced the ANC attendance (Table 1). Women who reported having a low income had a relatively low attendance (26.1%) as compared to those who reported a middle (71.4%) and high (100%) income. Moreover, women who spent less than 30 minutes to reach the health facility had relatively better attendance (88.9%) as compared to those who spent more than 30 minutes. Age, marital status, education level, religion, means of transport to the health facility, and awareness of ANC services did not have an influence on ANC attendance *(p value > .05)*.

### Qualitative results of factors influencing utilization of ANC services

Thirty-one women were interviewed, and three main themes of factors affecting ANC attendance emerged as summarised in Table 2. These factors include cultural practices and gender norms, poor communication between partners, and long waiting time at the ANC clinics. In quantitative analysis, none of these themed factors significantly influenced ANC attendance (Table 2).

**TABLE 2:** Themed Factors Contributing to Poor ANC Clinic Attendance

Factors	Total (%)	Attended ANC	P value
Yes	No	
Cultural practices and gender norms	17 (54.8)	5	12	0.103
Poor communication between spouses	12 (38.7)	3	9	
Long waiting time at the ANC clinics	2 (6.5)	2	0	

ANC = Antenatal Care

Slightly over half of the women (54.8%) reported that cultural practices and gender norms contributed to poor ANC attendance as exemplified by the following responses:

*“In our community men barely get involved in issues concerning pregnancy… even when we try to convince them, they refuse to accompany us to the clinic because they feel embarrassed.”*

*“According to my culture, my mother-in-law is the person responsible to care for my pregnancies… I did not have the opportunity to attend the clinic quite often during my pregnancy because my mother-in-law attended to most of my concerns, sometimes by using traditional means.”*

Twelve (38.7%) participants reported poor communication between partners as a reason for poor ANC attendance. One respondent mentioned that:

*“We barely talked with the father of my children about my pregnancy. Perhaps that is the reason he used to assign me a lot of duties to perform… If he was an understanding person and assisted in performing some of the household chores at least once in a while, then I think I could have time to attend the clinic”*

Only two participants (6.5%) reported that long waiting time at the ANC clinics was the reason for poor ANC clinic attendance. An interviewee mentioned that:

*“I engage in small-scale businesses as an entrepreneur. Long waiting time at the clinic consume the valuable time I could spend in generating income for my children.”*

Participants' Opinions on Improving Utilization of ANC Services Investing in Maternal Healthcare The government should establish a platform of collective responsibility with other partners in order to reach a satisfactory level of antenatal healthcare utilization. An interviewee mentioned that:

*“The government should encourage non-governmental health-based organizations to support antenatal healthcare programs by creating suitable regulations that will enable them to create well-planned and coordinated programs that support antenatal care with full community participation.”*

### Providing Affordable Services

Private sectors ought to make ANC services affordable so as to curtail the workload in public centers which provide services at a relatively cheaper cost. An interviewee mentioned that:

*“Private facilities also have to provide services at an affordable cost in order to reduce the overwhelming number of women attending public facilities.”*

### Improving Accessibility of Services

Equipped medical facilities should be increased in order to ease the accessibility of services. Moreover, increasing the number of health facilities should conform to the standard package of services to be provided by ANC facilities. A healthcare provider mentioned that:

*“We are often overloaded in the antenatal clinic because many women depend on this center for antenatal services, some of whom reside out of this administrative ward. Most of them spend a long time waiting for their turn to be attended to. In some cases, some become impatient and decide to leave. The government has to increase medical facilities and encourage investment so that all clients can have easy and equal access to quality antenatal services.”*

## DISCUSSION

Despite starting antenatal care at a later gestational age and having fewer than the recommended ANC visits, about 90% of pregnant women in Tanzania attend ANC clinics at least once during their pregnancies.^20^ This is low compared to 98% of women who receive ANC in developed countries.^21^ Although a significant proportion of the participants in this study were aware of ANC services provided at the health facilities, utilisation of the services remained low. The disproportionate attendance could be explained by low income and distant proximity to the healthcare facilities as presented in the current results.

Income has a significant bearing in healthcare-seeking behavior (HSB) with members of low-income communities being up to 6 times more likely to have inappropriate HSB as compared to members of high-income communities.^22,23^ In sub-Saharan Africa, low income has been linked to poor ANC attendance.^24^ Similarly, in this study, self-reported income status emerged as a significant determinant of ANC attendance, whereas women who reported having a low income had poor ANC clinic attendance. Furthermore, long waiting time at the ANC clinics was mentioned as a reason for low ANC utilization due to the impression of wasting valuable time in the case of women who engaged in income-generating activities. However, ANC services are relatively cheap in the current study setting^25^ thus, the lack of the concepts and practices of scientific medicine, and differences in beliefs and values within low-income communities might be an alternative explanation to the poor attendance.^26^ Concurring with the later argument, about 38% of the interviewees mentioned cultural practices and gender norms as the underlying contributors to poor ANC clinic attendance. Respondents revealed that there has been poor support and involvement of men in matters regarding reproductive health despite the ongoing campaigns encouraging the engagement of men in ANC.^27^ They also argued that some social-cultural practices and gender norms exempt men from supporting and accompanying their spouses to the ANC clinics. This is based on the belief among several tribes that experienced elderly women within the clan are the ones responsible for monitoring the health of young pregnant women; a strategy which has currently not been feasible due to rapid westernization in the urban communities. This subjects pregnant women to poor social support on matters of reproductive health.

Despite the increase in the number of health facilities across the country^28^ universal access to healthcare for those requiring MCH services is still limited. Partly, due to uneven distribution of health facilities, shortage of staff, and lack of equipment. Findings from this study revealed that 31.1% of the women spent over an hour reaching the ANC clinic. Moreover, longer durations taken to reach the health facility was linked to poor ANC attendance, with similar findings reported in other Sub-Saharan countries.^24^ Acceptable suppositions of the former finding could be a search for quality and cheap services. Evidence shows that women often bypass the closest facilities due to the poor quality of the services and are willing to travel a long distance in search of free services available in public hospitals.^29^ Corresponding to the later findings, Herman^26^ argued that living close to a health facility increased the probability of seeking health care.

Poor communication between spouses was mentioned as a contributor to poor ANC clinic attendance. In a patrilineal society as in the settings of our study, majority of household decisions are made mainly by husbands; and women are hardly the main decision-makers. Moreover, only 15% of wives are the main decision-makers regarding their health.^9^ Respondents argued that poor communication between partners on reproductive health matters caused men to be unaware of the women's health-seeking intentions, thus causing men to pause a tone of household duties on women while giving less priority to their health.

**FIGURE 3:**
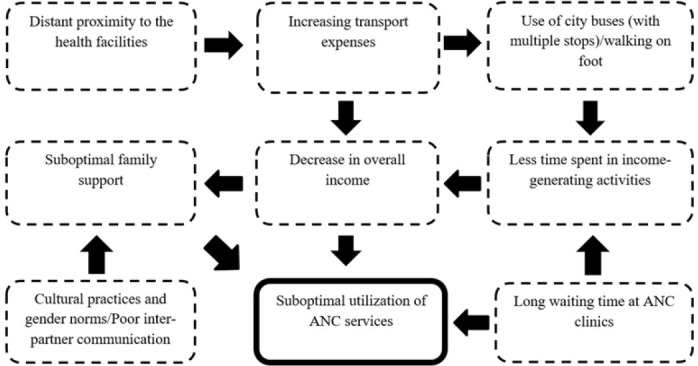
Proposed Web of Interlacing Events Leading to Poor ANC Attendance

A proposed relationship of the discussed findings has been illustrated in the web of events seen in Figure 3. Further studies will be needed to confirm or disprove this proposed hypothetical inter-relationship.

### Limitations

The purposive sampling technique that was employed limits the generalization of the findings to the subpopulation from which the sample was drawn. Furthermore, the small sample size limited by the study's inclusion criteria makes the quantitative component of the study findings prone to type II statistical error. Due to these limitations, we recommend additional studies that will address the aforementioned setbacks and extensively examine other variables that were not included in this study.

## CONCLUSION

Findings from this study demonstrate low utilization of ANC services among women living in Dodoma Urban District. Moreover, qualitative and quantitative evidence shows that self-reported income status, proximity to the health facility, cultural practices and gender norms, communication between partners, and service waiting time at the ANC clinics influence the utilization of ANC services. The study findings should guide policy formulation and interventions, targeting groups of women with low utilization of ANC services.

## RECOMMENDATIONS

The government and other stakeholders should focus on improving the coverage of ANC services in order to minimize the time spent reaching the health facilities. Furthermore, policies that govern the ANC user fees in both public and private sectors should be reviewed and refined by the respective authorities in order to suit women of low socioeconomic class. Policies should also focus on harmoniously addressing the sociocultural barriers to ANC utilization.

## References

[1] World Health Organization (WHO), UNICEF, UNFPA WB. Trends in maternal mortality 2010 - 2015, WHO. World Heal Organ. Published online 2015:92. http://www.who.int/reproductivehealth/publications/monitoring/maternal-mortality2015

[2] WHO, UNICEF, UNFPA, Group WB, Division UNP. Trends in Maternal Mortality: 2000 to 2017. Geneva, World Health Organization. Published 2019. Accessed October 3, 2021. https://data.worldbank.org/indicator/SH.STA.MMRT?end=2017&locations=TZ-KE-UG-BI-RW-SS&start=2000&type=shaded&view=chart

[3] Bwana VM, Rumisha SF, Mremi IR, Lyimo EP, Mboera LEG. Patterns and causes of hospital maternal mortality in Tanzania: A 10-year retrospective analysis. Zeeb H, ed. PLoS One. 2019;14(4):e0214807. doi:10.1371/journal.pone.0214807 30964909 PMC6456219

[4] McCarthy J, Maine D. A Framework for Analyzing the Determinants of Maternal Mortality. Stud Fam Plann. 1992;23(1):23. doi:10.2307/1966825 1557792

[5] World Health Organization. WHO Recommendation on Antenatal Care for a Positive Pregnancy Experience: Summary. Lancet. 2018; 387(10017):1–10. doi:10.1186/1742-4755-10-19.5 28079998

[6] UNICEF. The State of World's Children 2009.; 2009.

[7] Prata N, Passano P, Sreenivas A, Gerdts CE. Maternal mortality in developing countries: Challenges in scaling-up priority interventions. Women's Heal. 2010;6(2):311–327. doi:10.2217/whe.10.8 20187734

[8] Tessema ZT, Teshale AB, Tesema GA, Tamirat KS. Determinants of completing recommended antenatal care utilization in sub-Saharan from 2006 to 2018: evidence from 36 countries using Demographic and Health Surveys. BMC Pregnancy Childbirth. 2021;21(1):192. doi:10.1186/s12884-021-03669-w 33676440 PMC7937261

[9] Kearns Annie, Hurst Taylor, Caglia Jacquelyn LA. Focused antenatal care in Tanzania. Women Heal Initiat. 2014;(July):1–13. http://www.mhtf.org/wp-content/uploads/sites/32/2014/09/HSPH-Tanzania5.pdf

[10] Raru TB, Ayana GM, Zakaria HF, Merga BT. Association of Higher Educational Attainment on Antenatal Care Utilization Among Pregnant Women in East Africa Using Demographic and Health Surveys (DHS) from 2010 to 2018: A Multilevel Analysis. Int J Womens Health. 2022;Volume 14:67–77. doi:10.2147/IJWH.S350510 35140524 PMC8819274

[11] Rurangirwa AA, Mogren I, Nyirazinyoye L, Ntaganira J, Krantz G. Determinants of poor utilization of antenatal care services among recently delivered women in Rwanda; a population based study. BMC Pregnancy Childbirth. 2017;17(1):142. doi:10.1186/s12884-017-1328-2 28506265 PMC5430598

[12] Tessema ZT, Minyihun A. Utilization and Determinants of Antenatal Care Visits in East African Countries: A Multicountry Analysis of Demographic and Health Surveys. Adv Public Heal. 2021;2021:1–9. doi:10.1155/2021/6623009

[13] Nuamah GB, Agyei-Baffour P, Mensah KA, et al. Access and utilization of maternal healthcare in a rural district in the forest belt of Ghana. BMC Pregnancy Childbirth. 2019;19(1):6. doi:10.1186/s12884-018-2159-5 30612557 PMC6322319

[14] Nassoro MM, Chetto P, Chiwanga E, Lilungulu A, Bintabara D, Wambura J. Maternal Mortality in Dodoma Regional Referral Hospital, Tanzania. Int J Reprod Med. 2020;2020:1–6. doi:10.1155/2020/9082179 PMC7290896 32566647

[15] Straneo M, Fogliati P, Azzimonti G, Mangi S, Kisika F. Where Do the Rural Poor Deliver When High Coverage of Health Facility Delivery Is Achieved? Findings from a Community and Hospital Survey in Tanzania. PLoS One. 2014;9(12):e113995. doi:10.1371/JOURNAL.PONE.0113995 25460007 PMC4252065

[16] Maluka S, Chitama D, Dungumaro E, Masawe C, Rao K, Shroff Z. Contracting-out primary health care services in Tanzania towards UHC: how policy processes and context influence policy design and implementation. Int J Equity Health. 2018;17(1):118. doi:10.1186/s12939-018-0835-8 30286767 PMC6172831

[17] NordNordWest. Dodoma Municipal in der Region Dodoma, Tansania, Stand 2022. Published 2022. https://commons.wikimedia.org/wiki/File:Dodoma_Municipal_in_Dodoma_2022.svg

[18] Nowell LS, Norris JM, White DE, Moules NJ. Thematic Analysis. Int J Qual Methods. 2017;16(1):160940691773384. doi:10.1177/1609406917733847

[19] Ackerman N, Paolucci B. Objective and subjective income adequacy: Their relationship to perceived life quality measures. Social Indicators Research. doi:10.1007/BF00428859

[20] Maluka SO, Joseph C, Fitzgerald S, Salim R, Kamuzora P. Why do pregnant women in Iringa region in Tanzania start antenatal care late? A qualitative analysis. BMC Pregnancy Childbirth. 2020;20(1):126. doi:10.1186/s12884-020-2823-4 32093645 PMC7041254

[21] Zanconato G, Msolomba R, Guarenti L, Franchi M. Antenatal care in developing countries: The need for a tailored model. Semin Fetal Neonatal Med. 2006;11(1):15–20. doi:10.1016/j.siny.2005.10.002 16364704

[22] Latunji OO, Akinyemi OO. FACTORS INFLUENCING HEALTH-SEEKING BEHAVIOUR AMONG CIVIL SERVANTS IN IBADAN, NIGERIA. Ann Ibadan Postgrad Med. 2018;16(1):52–60. Accessed October 6, 2021. http://www.ncbi.nlm.nih.gov/pubmed/30254559 PMC6143883 30254559

[23] Li X, Deng L, Yang H, Wang H. Effect of socioeconomic status on the healthcare-seeking behavior of migrant workers in China. Wang J, ed. PLoS One. 2020;15(8):e0237867. doi:10.1371/journal.pone.0237867 32813702 PMC7444513

[24] Okedo-alex IN, Akamike IC, Ezeanosike OB, Uneke CJ. Determinants of antenatal care utilisation in sub-Saharan Africa : a systematic review. BMJ Open. Published online 2019:1–14. doi:10.1136/bmjopen-2019-031890 PMC6797296 31594900

[25] Shija AE, Msovela J, Mboera LEG. Maternal health in fifty years of Tanzania independence: Challenges and opportunities of reducing maternal mortality. Tanzan J Health Res. 2012;13(5):1–15. doi:10.4314/thrb.v13i5.5 26591990

[26] Herman M. The Poor: Their Medical Needs and the Health Services Available to Them. Ann Am Acad Pol Soc Sci. 1972;399:12–21.

[27] Osaki H, Sao SS, Kisigo GA, et al. Male engagement guidelines in antenatal care: unintended consequences for pregnant women in Tanzania. BMC Pregnancy Childbirth. 2021;21(1):720. doi:10.1186/s12884-021-04141-5 34702198 PMC8549379

[28] Ministry of Health and Social Welfare (MOHSW). Tanzania Services Provision Assessment Survey 2014-2015.; 2015.

[29] Escamilla V, Calhoun L, Winston J, Speizer IS. The Role of Distance and Quality on Facility Selection for Maternal and Child Health Services in Urban Kenya. J Urban Heal. 2018;95(1):1–12. doi:10.1007/s11524-017-0212-8 PMC5862698 29270709

